# Prognostic value of three different lymph node staging systems in the survival of patients with gastric cancer following D2 lymphadenectomy

**DOI:** 10.1007/s13277-015-4191-7

**Published:** 2016-02-24

**Authors:** Chen Jian-hui, Cai Shi-rong, Wu Hui, Chen Si-le, Xu Jian-bo, Zhai Er-tao, Chen Chuang-qi, He Yu-long

**Affiliations:** 1 Division of Gastrointestinal Surgery Center, The First Affiliated Hospital, Sun Yat-sen University, Guangzhou, 510080 China; 2 Gastric Cancer Center, Sun Yat-sen University, Guangzhou, 510080 China

**Keywords:** Log odds of positive lymph nodes, Lymph node ratio, Nodal system, Prognosis, Gastric cancer

## Abstract

The log odds of positive lymph nodes (LODDS) was defined as the log of the ratio between the number of positive lymph nodes and the number of negative lymph nodes, which is a novel and promising nodal staging system for gastric cancer. Here, we aimed to compare the prognostic effect of pN, lymph node ratio (LNR) and LODDS. The association between overall survival and pN, LNR and LODDS was retrospectively analysed. The discriminatory ability and monotonicity of gradients (linear trend *χ* ^2^ score), homogeneity ability (likelihood ratio test) and prognostic stratification ability (Akaike information criterion [AIC] and receiver operating characteristic [ROC] curve) were compared among three lymph node staging systems. The pN, LNR and LODDS were all identified as independent prognostic factors for gastric cancer patients in the multivariate analysis. LODDS showed the best prognostic performance (linear trend *χ* ^2^ score 266.743, likelihood ratio *χ* ^2^ test score 427.771, AIC value 5670.226, area under the curve (AUC) 0.793), followed by LNR and pN. In patients with different levels of retrieved lymph nodes (≤10, 11–14, 15–25 and >25), LODDS was the most powerful for prognostic prediction and discrimination of the heterogeneity among the subgroups. Significant differences in survival were observed among patients in different LODDS subgroups after being classified according to the pN and LNR classifications. LODDS appears to be a more powerful system for predicting the overall survival of gastric cancer patients, as compared to LNR and pN, and may serve as an alternative nodal staging system for gastric cancer.

## Introduction

According to the GLOBOCAN 2012 database, 952,000 new cases of gastric cancer and 723,000 cases of gastric cancer-related death are reported worldwide, which correspond to the total malignant cases of 8.5 and 10.1 %, respectively. Lymph node metastasis is known to be one of the most important prognostic factors for gastric cancer. Although the lymph node staging system, based on the extent of lymph node metastasis, was abandoned in the latest guideline of the Japanese Gastric Cancer Association (JGCA) [1], the prognostic ability of the lymph node staging system of the Union for International Cancer Control (UICC) remains controversial. Some authors indicated that the latest lymph node classification, as part of UICC staging, is influenced by the number of retrieved lymph nodes [2]. Moreover, certain studies suggested that the ratio-based lymph node system, which evaluates the ratio of metastatic lymph nodes and total retrieved lymph nodes, was theoretically superior to the involved number-based lymph node system, as the former system considers information of both metastatic and the total retrieved lymph nodes [3, 4]. The favourable results obtained from a comparison of survival with the ratio-based lymph node system suggest that this system may serve as an alternative to the traditional one. However, some studies indicated that patients with the same LNR staging had different survival outcomes, along with changes in the total number of retrieved lymph nodes, particularly when the LNR value was 0 or 1 [5].

Log odds of positive nodes (LODDS) is a novel, promising lymph node staging system and is defined as the log of the ratio of the number of positive lymph nodes and the total number of retrieved lymph nodes. The LODDS system has a better discrimination ability for patients without metastatic lymph nodes and has been considered as a more reliable method than the pN or LNR systems for prognostic evaluation in gastric cancer patients [6, 7]. Till now, there is no study to compare the prognostic significance of pN, LNR and LODDS systems together in Asian patients after D2 lymphadenectomy.

In the present study, we aimed to compare the prognostic performance of the pN, LNR and LODDS lymph node staging systems and to determine the most appropriate lymph node staging system for predicting overall survival in gastric cancer patients.

## Materials and methods

### Patients and surveillance

Between January 1994 and December 2008, all the cases diagnosed with gastric adenocarcinoma after radical surgery in the Department of Gastrointestinal Surgery of the First Affiliated Hospital, Sun Yat-sen University, China, were retrospectively analysed. The eligibility criteria were as follows: patients with gastric adenocarcinoma diagnosed via a histopathologic examination; patients receiving R0 resection and D2 lymphadenectomy, or extensive lymphadenectomy if necessary; patient death due to cancer and patients with complete follow-up data. The exclusion criteria were as follows: patients with a history of malignant tumours at other sites or gastric stump cancer. patients who were diagnosed with distant metastasis preoperatively or during the operation, patients who received neoadjuvant chemotherapy or radiochemotherapy and patients who died due to postoperative complications. In total, 935 patients were included in this study.

Written informed consent was obtained from all the patients preoperatively. The study was approved by the Ethics Committees of The First Affiliated Hospital of Sun Yat-sen University.

All the cases were followed up every 3 months in the first 2 years, every half year in the after 3 years and every year thereafter. Follow-up program consisted of physical examination, serum tumour markers, chest X-ray, annual gastroscope, abdominal CT scan or ultrasound every 6 months. The last follow-up was in December 2014. The median follow-up time was 56 months.

### Lymph node classifications

Lymph nodes were classified according to the seventh edition of the UICC/AJCC tumour-node-metastasis (TNM) system, based on the number of metastatic lymph nodes: N0, negative; N1, 1–2 positive lymph nodes; N2, 3–6 positive lymph nodes; N3, >6 positive lymph nodes. The LNR was defined as the ratio between the metastatic lymph nodes and total retrieved lymph nodes. The LNR ranged from 0 to 1 and was stratified at intervals of 0.1; it was used to compare the overall survival among each interval and among adjacent subgroups with similar survival outcomes. The LNR system was classified as follows: LNR0, 0; 0.01 < LNR1 ≤ 0.1; 0.1 < LNR2 ≤ 0.25 and LNR3 > 0.25. LODDS was defined as log ([pLN + 0.5] / [nLN + 0.5]), where pLN is the number of positive lymph nodes and nLN is the number of negative lymph nodes; 0.5 was added to both the numerator and denomination to avoid singularity. A similar method was performed to stratify LODDS, with an interval of 0.5. Thus, LODDS was classified as follows: LODDS1 ≤ −1.5, −1.5 < LODDS2 ≤ −1.0, −1.0 < LODDS3 ≤ 0.0 and LODDS4 > 0.0.

### Statistical analysis

The overall survival rate was calculated according to the life table method, and the log-rank test was used to assess statistical differences between groups. Survival curves were established using the Kaplan-Meier method. All the parameters that were statistically significant in the univariate analysis were included in the multivariate Cox proportional hazard model. With the Cox proportional hazard model, the likelihood ratio (*χ* ^2^) test was used to measure homogeneity between groups, whereas the Akaike information criterion (AIC) was adopted to minimise any potential bias when comparing different prognostic systems. The AIC was defined by a −2 log maximum likelihood +2, multiplied by the number of parameters in the model. The discriminatory ability and monotonicity of gradients were measured using the linear trend *χ* ^2^ test. The accuracy of the prognostic evaluation of different staging systems was compared using receiver operating characteristic (ROC) curve analysis and the area under the curve (AUC). The accepted level of statistical significance was defined as *P* < 0.05. The statistical analysis was performed using the SPSS 18.0 statistical package (SPSS Inc., Chicago, IL).

## Results

### Clinicopathological characteristics and survival analysis

In total, 935 patients were enrolled, including 636 men and 299 women, with a mean age of 57.5 years (range, 24–87 years). The gastric cancer was located in the lower part of the stomach (44.7 %) in most cases, followed by the upper portion (31.6 %), middle portion (21.7 %) and the entire stomach (2.0 %). Moreover, the average number of total retrieved lymph nodes was 24.9 per case (range, 0–140). The mean number of involved lymph nodes in our cohort was 4.61 (range, 0–124). Among 18.6 % of the patients, lymph node retrieval was insufficient. The 5-year survival rate of our cohort was 54.0 %, with a median overall survival time of 53.3 months (range, 1.3–148.4 months). Table 1 shows the demographics and survival results of our study.

**Table 1:** Clinicopathological characteristics and survival analysis of 935 patients with gastric cancer following radical gastrectomy

Variable	Patients (%)	5-year survival rate	*χ* ^2^ value (log-rank test)	*P* value
Age (years)			7.6767	0.006
<60	526 (56.3 %)	57.2 %		
≧60	409 (43.7 %)	49.9 %		
Sex			3.190	0.074
Male	636 (68.0 %)	52.0 %		
Female	299 (32.0 %)	58.2 %		
Tumour site			30.575	<0.001
Upper	295 (31.6 %)	43.1 %		
Middle	203 (21.7 %)	60.1 %		
Lower	418 (44.7 %)	60.0 %		
Whole	19 (2.0 %)	26.3 %		
Tumour size			79.694	<0.001
<5 cm	613 (65.6 %)	63.4 %		
≧5 cm	322 (34.4 %)	36.0 %		
Gross type			69.323	<0.001
Borrmann I + II	351 (37.5 %)	71.5 %		
Borrmann III + IV	584 (62.5 %)	43.4 %		
Degree of differentiation			12.898	<0.001
Well/moderately	340 (36.4 %)	61.1 %		
Pooly/undifferentiated	595 (63.6 %)	50.0 %		
Seventh T stage (UICC)			159.378	<0.001
T_1_	162 (17.3 %)	86.4 %		
T_2_	125 (13.4 %)	74.3 %		
T_3_	330 (35.3 %)	52.4 %		
T_4_	318 (34.0 %)	31.1 %		
Seventh N stage (UICC)			281.440	<0.001
N0	364 (38.9 %)	81.6 %		
N1	172 (18.4 %)	56.4 %		
N2	199 (21.3 %)	40.2 %		
N3	200 (21.4 %)	18.0 %		
LNR stage			368.726	<0.001
LNR0	364 (38.9 %)	81.6 %		
LNR1	143 (15.3 %)	63.6 %		
LNR2	197 (21.1 %)	42.6 %		
LNR3	231 (24.7 %)	14.3 %		
LODDS stage			428.720	<0.001
LODDS1	277 (29.6 %)	87.0 %		
LODDS2	170 (18.2 %)	65.9 %		
LODDS3	385 (41.2 %)	37.7 %		
LODDS4	103 (11.0 %)	7.0 %		
Number of LN retrieved			16.801	<0.001
<15	174 (18.6 %)	42.0 %		
≧15	761 (81.4 %)	56.7 %		

### Survival impact of pN, R stage and LODDS

**Fig. 1:**
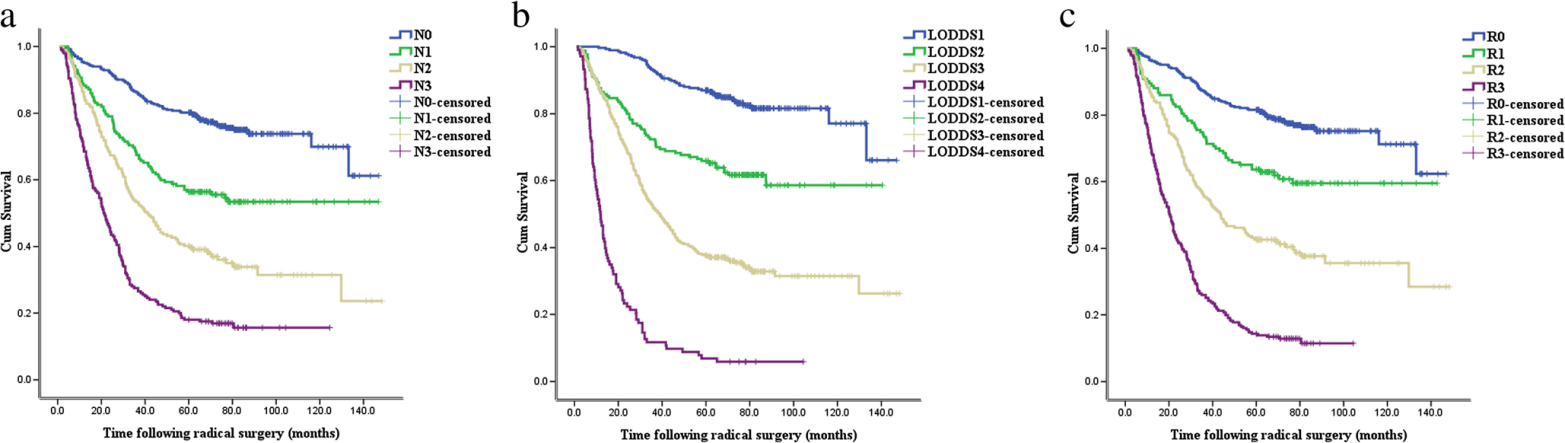
Kaplan-Meier analysis of overall survival (OS) for cases with gastric cancer according to different lymph node staging systems: **a** survival curves of patients according to the pN system, **b** survival curves of patients according to the LNR system and **c** survival curves of patients according to the LODDS system

The survival curves of patients according to pN, LNR and LODDS were all significantly different (Fig. 1a–c) (all, *P* < 0.001). In the univariate analysis, age, tumour location, tumour size, gross type, histological type, number of retrieved lymph nodes, pN, LNR and LODDS were significantly correlated with overall survival (Table 2). All the potential prognostic factors identified in the univariate analysis were included in the multivariate analysis. All the three lymph node staging systems (pN, LNR and LODDS) were identified as significantly independent prognostic factors of overall survival in our cohort (Table 3).

**Table 2:** Univariate analysis of overall survival in gastric cancer

Parameters	*χ* ^2^ value	Hazard ratio	95 % CI^a^	*P* value
Age	7.619	1.293	1.077–1.551	0.006
Gender	3.183			0.074
Tumour location	29.540			<0.001
Upper				
Middle	12.393	0.632	0.489–0.816	<0.001
Lower	17.246	0.645	0.525–0.794	<0.001
Whole	3.480	1.679	0.974–2.894	0.062
Tumour size	75.377	2.251	1.874–2.703	<0.001
Gross type	65.028	2.393	1.936–2.959	<0.001
Histological type	12.735	1.430	1.175–1.741	<0.001
T stage	134.757			<0.001
T_1_				
T_2_	4.773	1.716	1.057–2.787	0.029
T_3_	42.484	3.645	2.470–5.377	<0.001
T_4_	91.655	6.449	4.403–9.446	<0.001
Insufficient retrieved lymph nodes	16.484	0.637	0.512–0.792	<0.001
pN	101.762			<0.001
pN_0_ (reference)				
pN_1_	11.413	1.716	1.254–2.347	0.001
pN_2_	37.078	2.461	1.842–3.289	<0.001
pN_3_	94.807	4.255	3.161–5.648	<0.001
rN	191.740			<0.001
rN_0_ (reference)				
rN_1_	20.962	2.407	1.653–3.506	<0.001
rN_2_	70.592	4.050	2.922–5.612	<0.001
rN_3_	174.151	12.756	8.739–18.618	<0.001
LODDS	427.771			<0.001
LODDS1 (reference)				
LODDS2	27.166	2.697	1.857–3.917	<0.001
LODDS3	123.286	5.777	4.239–7.874	<0.001
LODDS4	272.255	19.594	13.761–27.900	<0.001

*CI* ^a^ confidence interval

**Table 3:** Multivariate analysis of overall survival in gastric cancer for the different lymph node staging systems

Parameters	Multivariate analysis 1			Multivariate analysis 2			Multivariate analysis 3		
	HR	95 % CI^a^	*p* Value	HR	95 % CI^a^	*p* Value	HR	95 % CI^a^	*P* value
Age	1.369	1.139–1.646	0.001	1.409	1.171–1.695	<0.001	1.340	1.1141.612	0.002
Gender									
Tumour location									
Upper									
Middle									
Lower									
Whole									
Tumour size	1.320	1.087–1.603	0.005	1.257	1.034–1.527	0.021			
Gross type	1.292	1.024–1.630	0.031	1.346	1.069–1.685	0.012	1345	1.073–1.686	0.010
Histological type									
T stage									<0.001
T_1_ (reference)									
T_2_	1.093	0.664–1.799	0.728	1.070	0.648–1.765	0.792	1.029	0.625–1.693	0.911
T_3_	1.761	1.150–2.694	0.009	1.722	1.127–2.633	0.012	1.818	1.197–2.759	0.005
T_4_	2.224	1.440–3.433	<0.001	2.173	1.409–3.350	<0.001	2.363	1.550–3.603	<0.001
Insufficient retrieved lymph nodes	0.488	0.389–0.612	<0.001	0.622	0.499–0.776	<0.001	0.748	0.597–0.938	<0.001
pN			<0.001						
pN_0_ (reference)									
pN_1_	1.697	1.241–2.320	0.001						
pN_2_	2.610	1.951–3.491	<0.001						
pN_3_	4.987	3.704–6.714	<0.001						
rN						<0.001			
rN_0_ (reference)									
rN_1_				1.617	1.142–2.29	0.007			
rN_2_				2.486	1.843–3.353	<0.001			
rN_3_				5.702	4.280–7.596	<0.001			
LODDS									<0.001
LODDS1 (reference)									
LODDS2							2.144	1.457–3.153	<0.001
LODDS3							3.840	2.765–5.333	<0.001
LODDS4							12.035	8.234–17.590	<0.001

*CI* ^a^ confidence interval

### Comparison of prognostic ability among pN, R stage and LODDS

To identify the most appropriate system of representing lymph node involvement for the evaluation of overall survival in gastric cancer patients, we adopted the linear trend *χ* ^2^ score to evaluate the discriminatory ability and monotonicity of gradients, the likelihood ratio (*χ* ^2^) test to assess homogeneity ability and AIC value and ROC curve to compare the prognostic ability among the systems. We observed that LODDS had the highest linear trend *χ* ^2^ score and likelihood ratio (*χ* ^2^) test score, lowest AIC value and largest AUC (Table 4). Therefore, we considered that the LODDS was superior to LNR and pN.

**Table 4:** Prognostic ability comparison among the different lymph node staging systems for gastric cancer

	Linear trend *χ* ^2^ score	Likelihood ratio *χ* ^2^ test	AIC value	ROC area
Seventh UICC pN	215.021	280.826	5769.037	0.767
rN	262.177	367.908	5706.294	0.793
LODDS	266.743	427.771	5670.226	0.793

**Fig. 2:**
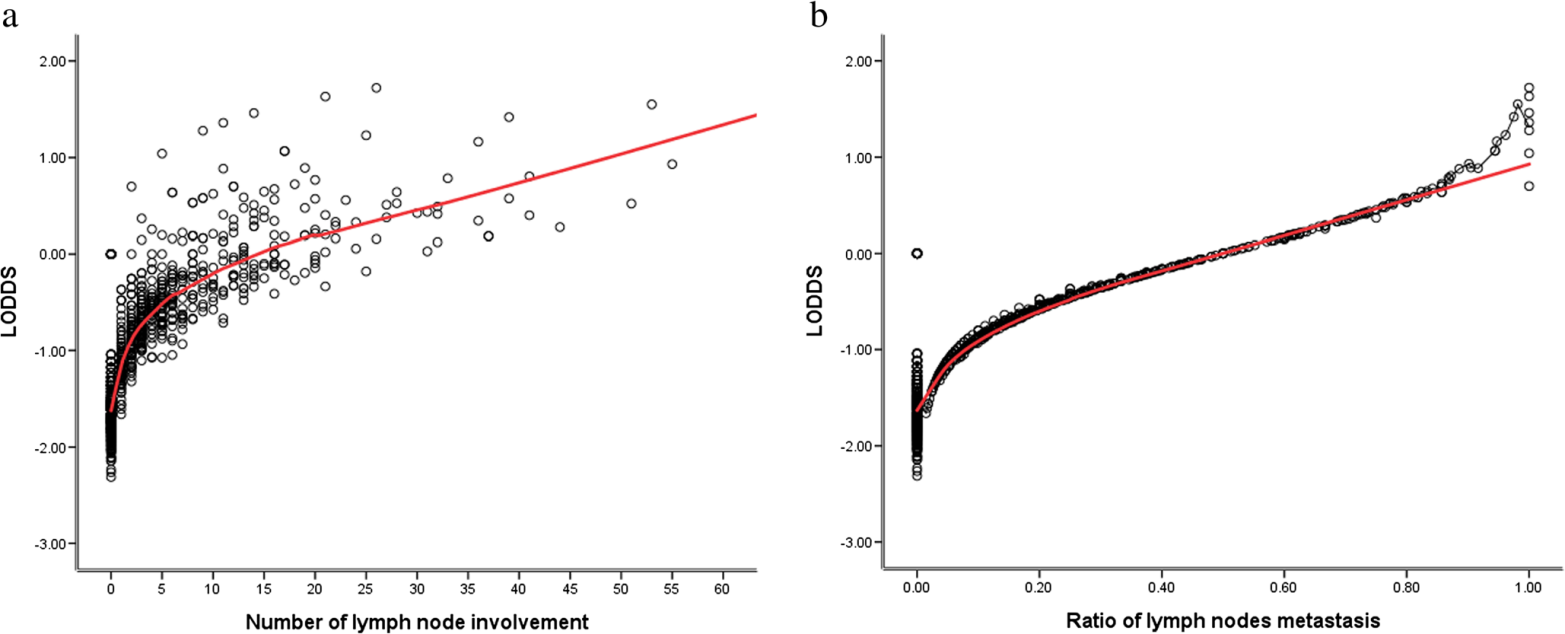
Scatter plots of the relationship between LODDS and the number or ratio of lymph nodes involved: **a** scatter plots of the relationship between LODDS and the number of metastatic lymph nodes and **b** scatter plots of the relationship between LODDS and LNR

To elucidate the reason for the superiority of LODDS compared to pN and LNR, we created scatter plots of the relationship between LODDS and the number or ratio of lymph node involvement. As shown in Fig. 2a, b, the LODDS value increased with the number and the ratio of metastatic lymph nodes, indicating close relationships between LODDS and pN, as well as LNR. However, these correlations were not linear. When the number of lymph nodes involved was ≤10 or when the ratio of lymph node metastasis was <0.2, the curves of pN and LNR increased at a slower rate as compared to the LODDS, indicating that LODDS could be superior to pN and LNR in the prediction of long-term overall survival of the cases mentioned above. Moreover, when the ratio of lymph node metastasis was 0 or 1, the LODDS score was heterogeneous, indicating that the LODDS system had the potential to indicate different survival outcomes for patients with the same LNR stage, especially for cases with an LNR score of 0 or 1.

### Correlation among the total number of retrieved lymph nodes, pN, LNR and LODDS

A Pearson test was conducted to evaluate the correlation of the total number of retrieved lymph nodes with the pN, LNR and LODDS systems. A strong relationship was observed between the total number of retrieved lymph nodes and the number or ratio of metastatic lymph nodes (*r* = 0.214 and 0.077, *P* < 0.001 and 0.018, respectively). However, there was no association between the number of retrieved lymph nodes and LODDS (*r* = −0.038, *P* = 0.251). The results of Pearson analysis indicated that LODDS was positively correlated with pN and LNR (*r* = 0.868 and 0.888, *P* < 0.001 and <0.001, respectively), whereas a strong relationship was present between pN and LNR (*r* = 0.938, *P* < 0.001).

### Evaluation of the prognostic value of different lymph node staging systems with different levels of retrieved lymph nodes

To assess the prognostic abilities of pN, LNR and LODDS with different levels of retrieved lymph nodes, all the patients were divided into four groups according to the number of retrieved lymph nodes: group 1 (≤10, *n* = 93), group 2 (11–14, *n* = 81), group 3 (15–25, *n* = 405) and group 4 (>25, *n* = 356). As shown in Table 5, pN, LNR and LODDS showed prognostic stratification abilities among the different subgroups. We further compared the prognostic performance among the groups. We noted that in all four groups, the LODDS system had the highest linear trend *χ* ^2^ score, likelihood ratio (*χ* ^2^) test score and lowest AIC value, followed by the LNR system and pN system. Similarly, the LODDS system had the largest AUC in group 1 and group 3. However, in group 2 and group 4, the LNR system had the largest AUC, followed by the LODDS and pN systems (Table 6).

**Table 5:** Evaluation of the prognostic value of the different lymph node systems with different levels of retrieved lymph nodes

	Number	5-year survival rate	*χ* ^2^ value	*P* value
Retrieved LN (≦10)	93			
Seventh UICC pN staging			9.792	0.020
pN_0_	47 (50.5 %)	61.7 %		
pN_1_	25 (26.9 %)	40.0 %		
pN_2_	18 (19.4 %)	33.3 %		
pN_3_	3 (3.2 %)	0.0 %		
pN ratio			39.029	<0.001
rN_0_	47 (50.5 %)	74.5 %		
rN_1_	5 (5.4 %)	40.0 %		
rN_2_	15 (16.1 %)	33.3 %		
rN_3_	26 (28.0 %)	11.5 %		
LODDS			64.853	<0.001
LODDS1	4 (4.3 %)	100.0 %		
LODDS2	42 (45.2 %)	73.8 %		
LODDS3	35 (37.6 %)	28.6 %		
LODDS4	12 (12.9 %)	0.0 %		
Retrieved LN (11–14)	81			
Seventh UICC pN staging			33.848	<0.001
pN_0_	34 (42.0 %)	91.2 %		
pN_1_	18 (22.2 %)	27.8 %		
pN_2_	16 (19.8 %)	25.0 %		
pN_3_	13 (16.0 %)	23.1 %		
pN ratio			61.503	<0.001
rN_0_	34 (42.0 %)	91.2 %		
rN_1_	9 (11.1 %)	55.6 %		
rN_2_	18 (22.2 %)	33.3 %		
rN_3_	20 (24.7 %)	5.0 %		
LODDS			66.012	<0.001
LODDS1	3 (3.7 %)	100.0 %		
LODDS2	31 (38.3 %)	90.3 %		
LODDS3	34 (42.0 %)	35.3 %		
LODDS4	13 (16.0 %)	0.0 %		
Retrieved LN (15–25)	405			
Seventh UICC pN staging			101.302	<0.001
pN_0_	164 (40.5 %)	79.9 %		
pN_1_	77 (40.5 %)	57.1 %		
pN_2_	104 (25.7 %)	41.3 %		
pN_3_	60 (14.8 %)	18.3 %		
pN ratio			117.273	<0.001
rN_0_	164 (40.5 %)	79.9 %		
rN_1_	62 (15.3 %)	61.3 %		
rN_2_	96 (23.7 %)	47.9 %		
rN_3_	83 (20.5 %)	16.9 %		
LODDS			135.377	<0.001
LODDS1	147 (36.3 %)	85.7 %		
LODDS2	48 (11.9 %)	52.1 %		
LODDS3	175 (43.2 %)	42.3 %		
LODDS4	35 (8.6 %)	11.4 %		
Retrieved LN (>25)	356			
Seventh UICC pN staging			150.634	<0.001
pN_0_	119 (33.4 %)	84.0 %		
pN_1_	53 (14.9 %)	71.6 %		
pN_2_	60 (16.9 %)	46.7 %		
pN_3_	124 (34.8 %)	17.7 %		
pN ratio			160.727	<0.001
rN_0_	119 (33.4 %)	84.0 %		
rN_1_	69 (19.4 %)	68.1 %		
rN_2_	67 (18.8 %)	40.3 %		
rN_3_	101 (28.4 %)	13.9 %		
LODDS			173.033	<0.001
LODDS1	123 (34.6 %)	87.8 %		
LODDS2	49 (13.8 %)	57.1 %		
LODDS3	141 (39.6 %)	34.8 %		
LODDS4	43 (12.1 %)	7.0 %

**Table 6:** Comparison of prognostic value of the different pN staging systems with different levels of retrieved lymph nodes for gastric cancer

	Linear trend *χ* ^2^ score	Likelihood ratio *χ* ^2^ test	AIC value	ROC area
Retrieved LN (≦10)				
Seventh UICC pN	9.577	9.776	410.023	0.669
rN	28.113	38.959	384.198	0.792
LODDS	29.657	64.653	370.819	0.804
Retrieved LN (11–14)				
Seventh UICC pN	24.720	33.769	306.713	0.829
rN	39.795	61.343	292.089	0.889
LODDS	49.661	65.832	290.830	0.877
Retrieved LN (15–25)				
Seventh UICC pN	80.198	101.110	2109.785	0.747
rN	88.779	117.045	2099.359	0.760
LODDS	95.421	135.126	2082.645	0.763
Retrieved LN (>25)				
Seventh UICC pN	111.970	150.229	1851.248	0.809
rN	113.450	160.300	1848.628	0.813
LODDS	114.897	172.573	1840.384	0.810

### Survival analysis of the pN and LNR subgroups according to the LODDS stratification

To evaluate the survival impact of the LODDS stratification as per the pN and LNR systems, all the distinct prognostic cohorts stratified according to the pN or LNR systems were analysed based on different LODDS stratifications. We observed that the LODDS could discriminate among N0, N1, N2 and N3, regardless of whether the pN or LNR system was used, suggesting that LODDS had excellent prognostic discriminatory ability to assess the current lymph node system. All the survival data and statistical results were shown in Table 7.

**Table 7:** Survival analysis of pN stage and rN systems according to the LODDS staging system

	Number	5-year survival rate	*χ* ^2^ value	*P* value
UICC pN				
UICC pN_0_			22.895	<0.001
LODDS1	267 (73.4 %)	89.9 %		
LODDS2	97 (26.6 %)	67.0 %		
UICC pN_1_			28.892	<0.001
LODDS1	10 (5.8 %)	90.0 %		
LODDS2	68 (39.3 %)	64.7 %		
LODDS3	94 (54.3 %)	46.8 %		
LODDS4	1 (0.6 %)	0.0 %		
UICC pN_2_			11.004	0.004
LODDS2	6 (3.0 %)	83.3 %		
LODDS3	184 (92.9 %)	40.2 %		
LODDS4	8 (4.1 %)	12.5 %		
UICC pN_3_			17.658	<0.001
LODDS3	106 (53.0 %)	26.8 %		
LODDS4	94 (47.0 %)	9.6 %		
rN				
rN_0_			22.895	<0.001
LODDS1	267 (73.4 %)	89.9 %		
LODDS2	97 (26.6 %)	67.0 %		
rN_1_			9.265	0.010
LODDS1	10 (6.8 %)	90.0 %		
LODDS2	74 (50.7 %)	69.2 %		
LODDS3	62 (42.5 %)	50.4 %		
rN_2_			6.366	0.012
LODDS2	19 (9.7 %)	73.7 %		
LODDS3	177 (90.3 %)	40.1 %		
rN_3_			22.961	<0.001
LODDS3	126 (55.0 %)	17.6 %		
LODDS4	103 (45.0 %)	9.7 %		

## Discussion

Information on lymph node involvement in gastric cancer has a great clinical impact on treatment decisions and survival assessment. The number-based lymph node staging system of UICC classification (seventh edition) was widely used, although a principle flaw is that the prognostic accuracy is influenced by the number of total retrieved lymph nodes [8, 9]. The most recent NCCN guideline recommends that D2 lymphadenectomy should be used to remove an adequate number of lymph nodes (no less than 15); however, some studies reported that ≥15 lymph nodes are harvested in only 29 and 60.2 % of cases in the USA [10] and China [11]. Moreover, the optimal number of retrieved lymph nodes to avoid stage migration remains unclear in some studies [12, 13]. Hence, in cases where the number of tested lymph nodes was insufficient, the staging migration phenomenon may occur.

The LNR system considers both the information of nodal involvement and total lymph nodes tested and can theoretically overcome the limitations of number-based nodal system. Most authors indicated that LNR was superior to the pN system due to the presence of a larger power for minimising staging migration [14]. A recent large cohort study (nearly 9000 cases) on gastric cancer in Korea [15] concluded that the LNR system is a better alternative for predicting long-term survival and compensating for the stage migration effect. However, due to some limitations, it cannot be considered as an alternative to the current pN staging. First, there is no difference among the patients with negative lymph nodes between the pN and LNR systems, indicating that LNR does not have the ability to distinguish the difference in survival in pN0 patients. Moreover, the cut-off value of LNR reported in several studies is varied [14, 16, 17]. In addition, the patients in the same LNR stage may have different survival outcomes, with a different number of tested lymph nodes [18].

LODDS—a novel system for assessing the status of lymph node involvement—is an alternative option for the evaluation of metastatic lymph nodes to predict overall survival; however, this system has only been investigated in cases of colon cancer and breast cancer. Following the report of Sun [6] who emphasised the favourable prognostic impact of LODDS in gastric cancer, some studies in China and North America have validated its use for prognostic purposes. Another study [11] proposed a new tumour-LODDS-metastasis (TLM) classification, which was based on the LODDS system, and was superior to the TNM and tumour-ratio-metastasis (TRM) classifications in the prognostic assessment of cases following D2 lymphadenectomy. Some studies did not note any superiority of LODDS over the pN or LNR systems [10, 19]. Yu [20] indicated the LODDS system may reflect a false survival outcome for patients with gastric cancer, with a reduced true hazard ratio of the N status against survival. An ideal lymph node staging system should satisfy three conditions [21]: decreased patient survival with increasing stage (monotonicity), similar survival within a group (homogeneity) and difference in survival between groups (distinctiveness). To our knowledge, the present study is the first to compare the above three conditions among the pN, LNR and LODDS systems. In multivariate Cox regression analysis in the present study, the pN, LNR and LODDS systems were all found to be independent parameters for overall survival. Moreover, the LODDS system had the highest linear trend *χ* ^2^ score, likelihood ratio (*χ* ^2^) test score, largest AUC and lowest AIC value, indicating that the LODDS system was superior to both the pN system and LNR system.

Theoretically, the LODDS system was superior to the pN system in minimising stage migration, particularly in cases where the number of analysed lymph nodes was insufficient [22]. We further interpret that this novel system had a remarkable robustness in terms of the prognostic effect and discrimination ability at different levels of tested lymph nodes. As shown in Table 5, when the number of retrieved lymph nodes was insufficient (≤10 or 11–14), the LODDS system had the best performance in homogeneity, discriminatory ability, monotonicity of gradients and accuracy of the prognosis evaluation, followed by the LNR system and pN system. Moreover, in the present study, the pN and LNR systems were positively correlated with the number of retrieved lymph nodes. However, unlike that reported in the study of Qiu [11], we found that there was no relationship between the number of tested lymph nodes and the LODDS system, indicating that the LNR and pN systems were affected by the total number of tested lymph nodes, whereas the LODDS system was unaffected. Moreover, Aurello [23] reported that the LODDS system was a function of the number of negative lymph nodes, whereas the LNR system was a function of the number of total retrieved lymph nodes; hence, theoretically, the LODDS system was superior to the LNR system. Jiao [24] explained that cases with an increasing number of tested lymph nodes may reflect better survival, particularly in patients with node-negative gastric cancer. For patients with an LNR value 0 or 1, the LNR system had no discriminatory power. As shown in Fig. 2b, the LODDS system can distinguish patients with an LNR value 0 or 1, which accounted for 39.6 % of the total number of patients. Moreover, using the log-rank test, we noted that LODDS had the ability to distinguish between patients with different pN or LNR systems into distinct prognostic groups. This evidence strongly suggests that the LODDS system may be the most reliable method for lymph node classification in gastric cancer.

Our study had certain limitations. First, all data was obtained from a single institution, and this may not reflect the status in other centres in China. Moreover, the method of calculation in the LODDS system was complicated, which may limit the clinical application of the LODDS system.

In conclusion, the pN, LNR and LODDS systems appear to be independent prognostic factors, as determined by the multivariate analysis. Although the LODDS system was more complex, it showed prognostic superiority over both the pN and LNR systems in our study. It can discriminate survival differences of gastric cancer patients with negative lymph nodes and an insufficient number of retrieved lymph nodes. Hence, the LODDS system should be considered as a novel and promising lymph node staging system for gastric cancer in the future.

## References

[1] Japanese Gastric Cancer Association (2010). Japanese gastric cancer treatment guidelines (ver. 3). Gastric Cancer.

[2] Kutlu OC, Watchell M, Dissanaike S (2015). Metastatic lymph node ratio successfully predicts prognosis in western gastric cancer patients. Surg Oncol.

[3] Wong J, Rahman S, Saeed N, Lin HY, Almhanna K, Shridhar R (2013). Prognostic impact of lymph node retrieval and ratio in gastric cancer: a U.S. single center experience. J Gastrointest Surg.

[4] Zhang BY, Yuan J, Cui ZS, Li ZW, Li XH, Lu YY (2014). Evaluation of the prognostic value of the metastatic lymph node ratio for gastric cancer. Am J Surg.

[5] Wang X, Appleby DH, Zhang X, Gan L, Wang JJ, Wan F (2013). Comparison of three lymph node staging schemes for predicting outcome in patients with gastric cancer. Br J Surg.

[6] Sun Z, Xu Y, de Li M, Wang ZN, Zhu GL, Huang BJ (2010). Log odds of positive lymph nodes: a novel prognostic indicator superior to the number-based and the ratio-based N category for gastric cancer patients with R0 resection. Cancer.

[7] Calero A, Escrig-Sos J, Mingol F, Arroyo A, Martinez-Ramos D, de Juan M (2015). Usefulness of the log odds of positive lymph nodes to predict and discriminate prognosis in gastric carcinomas. J Gastrointest Surg.

[8] Wu XJ, Miao RL, Li ZY, Bu ZD, Zhang LH, Wu AW (2015). Prognostic value of metastatic lymph node ratio as an additional tool to the TNM stage system in gastric cancer. Eur J Surg Oncol.

[9] Sun Z, Zhu GL, Lu C, Guo PT, Huang BJ, Li K (2009). The impact of N-ratio in minimizing stage migration phenomenon in gastric cancer patients with insufficient number or level of lymph node retrieved: results from a Chinese mono-institutional study in 2159 patients. Ann Oncol.

[10] Liu H, Deng J, Zhang R, Hao X, Jiao X, Liang H (2013). The RML of lymph node metastasis was superior to the LODDS for evaluating the prognosis of gastric cancer. Int J Surg.

[11] Qiu MZ, Qiu HJ, Wang ZQ, Ren C, Wang DS, Zhang DS (2012). The tumor-log odds of positive lymph nodes-metastasis staging system, a promising new staging system for gastric cancer after D2 resection in China. PLoS One.

[12] Shen Z, Ye Y, Xie Q, Liang B, Jiang K, Wang S. Effect of the number of lymph nodes harvested on the long-term survival of gastric cancer patients according to tumor stage and location: a 12-year study of 1,637 cases. Am J Surg 2015; 16: S0002-9610(15)00237-8. 10.1016/j.amjsurg.2015.01.029 26070380

[13] Biondi A, D’Ugo D, Cananzi FC, Papa V, Borasi A, Sicoli F (2015). Does a minimum number of 16 retrieved nodes affect survival in curatively resected gastric cancer?. Eur J Surg Oncol.

[14] Deng J, Zhang R, Wu L, Zhang L, Wang X, Liu Y (2015). Superiority of the ratio between negative and positive lymph nodes for predicting the prognosis for patients with gastric cancer. Ann Surg Oncol.

[15] Kong SH, Lee HJ, Ahn HS, Kim JW, Kim WH, Lee KU (2012). Stage migration effect on survival in gastric cancer surgery with extended lymphadenectomy: the reappraisal of positive lymph node ratio as a proper N-staging. Ann Surg.

[16] Lorenzon L, Mercantini P, Ferri M, La Torre M, Sparagna A, Balducci G (2014). Lymph-node ratio classification strongly correlates with cancer survivals of patients who underwent r0 resection for gastric cancer with more than 15 nodes harvested. Eur Surg Res.

[17] Taghizadeh-Kermani A, Yahouiyan SZ, AliAkbarian M, Seilanian TM (2014). Prognostic significance of metastatic lymph node ratio in patients with gastric cancer: an evaluation in north-East of iran. Iran J Cancer Prev.

[18] Wang J, Hassett JM, Dayton MT, Kulaylat MN (2008). The prognostic superiority of log odds of positive lymph nodes in stage III colon cancer. J Gastrointest Surg.

[19] Smith DD, Nelson RA, Schwarz RE (2014). A comparison of five competing lymph node staging schemes in a cohort of resectable gastric cancer patients. Ann Surg Oncol.

[20] Yu C, Ying Z, Chen W (2011). The impact of calculation procedure on the hazard ratio of N stages. Ann Surg Oncol.

[21] Rice TW, Rusch VW, Ishwaran H, Blackstone EH, Worldwide Esophageal Cancer Collaboration (2010). Cancer of the esophagus and esophagogastric junction: data-driven staging for the seventh edition of the American Joint Committee on Cancer/International Union Against Cancer Cancer Staging Manuals. Cancer.

[22] Spolverato G, Ejaz A, Kim Y, Squires MH, Poultsides G, Fields RC, et al. Prognostic performance of different lymph node staging systems after curative intent resection for gastric adenocarcinoma. Ann Surg. 2015; [Epub ahead of print]. 10.1097/SLA.0000000000001040 25563867

[23] Aurello P, Petrucciani N, Nigri GR, La Torre M, Magistri P, Tierno S (2014). Log odds of positive lymph nodes (LODDS): what are their roles in the prognostic assessment of gastric adenocarcinoma?. J Gastrointest Surg.

[24] Jiao XG, Deng JY, Zhang RP (2014). Prognostic value of number of examined lymph nodes in patients with node-negative gastric cancer. World J Gastroenterol.

